# Supplementary material for: The Role of Basic Psychological Needs in the Adoption of Healthy Habits by Adolescents
Source: Behav Sci (Basel). 2023 Jul 14;13(7):592. doi: 10.3390/bs13070592 (PMC10376858; doi:10.3390/bs13070592)
Supplement: Supplementary file 1 [file behavsci-13-00592-s001.zip › Supplementary Figure S1.pdf]

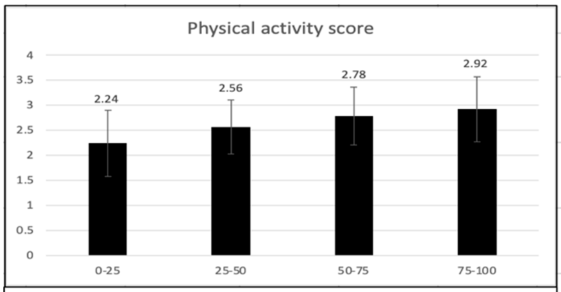

0-25 vs 25-50: Mean Diff: -0.319; p<0.001; 95% CI: -0.49; -0.15
0-25 vs 50-75: Mean Diff: -0.540; p<0.001; 95% CI: -0.72; -0.36
0-25 vs 75-100: Mean Diff: -0.675; p<0.001; 95% CI: -0.85; -0.51
25-50 vs 50-75: Mean Diff: -0.221; p=0.004; 95% CI: -0.39; -0.05
25-50 vs 75-100: Mean Diff: -0.356; p<0.001; 95% CI: -0.51; -0.20
50-75 vs 75-100: Mean Diff: -0.136; p=0.188; 95% CI: -0.30; 0.03

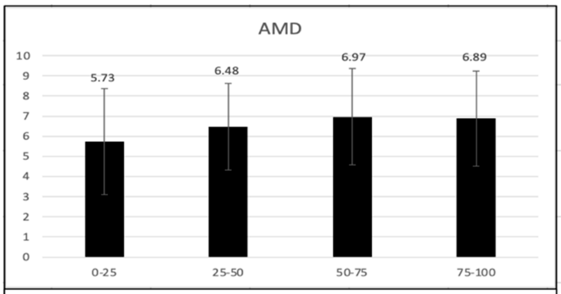

0-25 vs 25-50: Mean Diff: -0.75; p=0.020; 95% CI: -1.42; -0.08
0-25 vs 50-75: Mean Diff: -1.24; p<0.001; 95% CI: -1.96; -0.52
0-25 vs 75-100: Mean Diff: -1.15; p<0.001; 95% CI: -1.81; -0.49
25-50 vs 50-75: Mean Diff: -0.49; p=0.289; 95% CI: -1.15; 0.17
25-50 vs 75-100: Mean Diff: -0.41; p=0.429; 95% CI: -1.00; 0.19
50-75 vs 75-100: Mean Diff: 0.09; p=1.000; 95% CI: -0.56; 0.74

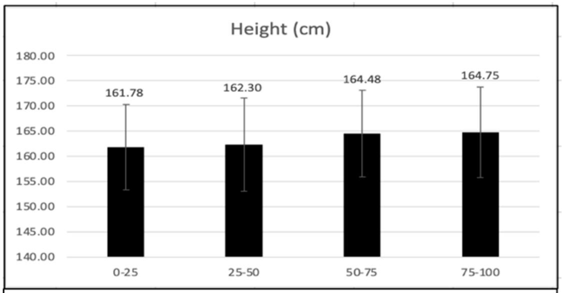

0-25 vs 25-50: Mean Diff: -0.527; p=1.000; 95% CI: -3.05; 1.99
0-25 vs 50-75: Mean Diff: -2.705; p=0.050; 95% CI: -5.41; -0.01
0-25 vs 75-100: Mean Diff: -2.974; p=0.010; 95% CI: -5.46; -0.49
25-50 vs 50-75: Mean Diff: -2.178; p=0.122; 95% CI: -4.65; 0.30
25-50 vs 75-100: Mean Diff: -2.447; p=0.023; 95% CI: -4.68; -0.21
50-75 vs 75-100: Mean Diff: -0.269; p=1.000; 95% CI: -2.71; 2.17

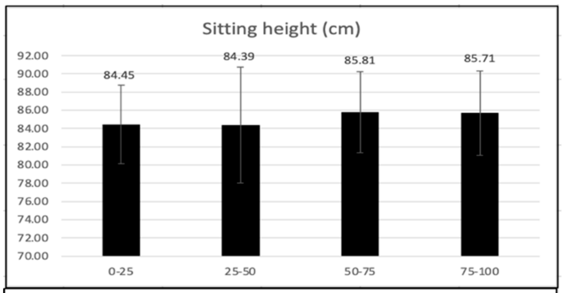

0-25 vs 25-50: Mean Diff: -0.056; p=1.000; 95% CI: -1.39; 1.50
0-25 vs 50-75: Mean Diff: -1.359; p=0.122; 95% CI: -2.91; -0.19
0-25 vs 75-100: Mean Diff: -1.257; p=0.117; 95% CI: -2.68; 0.16
25-50 vs 50-75: Mean Diff: -1.415; p=0.051; 95% CI: -2.83; 0.01
25-50 vs 75-100: Mean Diff: -1.313; p=0.040; 95% CI: -2.59; -0.03
50-75 vs 75-100: Mean Diff: 0.102; p=1.000; 95% CI: -1.29; 1.50

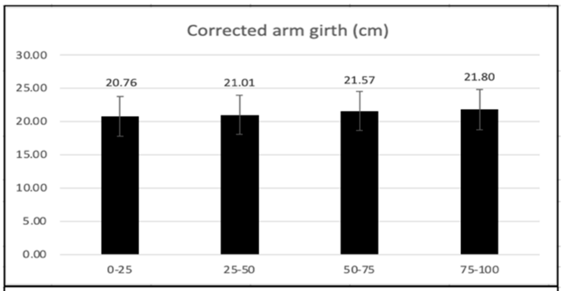

0-25 vs 25-50: Mean Diff: -0.254; p=1.000; 95% CI: -1.10; 0.59
0-25 vs 50-75: Mean Diff: -0.809; p=0.111; 95% CI: -1.72; 0.10
0-25 vs 75-100: Mean Diff: -1.045; p=0.006; 95% CI: -1.88; -0.21
25-50 vs 50-75: Mean Diff: -0.555; p=0.464; 95% CI: -1.39; 0.28
25-50 vs 75-100: Mean Diff: -0.791; p=0.032; 95% CI: -1.54; -0.04
50-75 vs 75-100: Mean Diff: -0.236; p=1.000; 95% CI: -1.05; 0.58

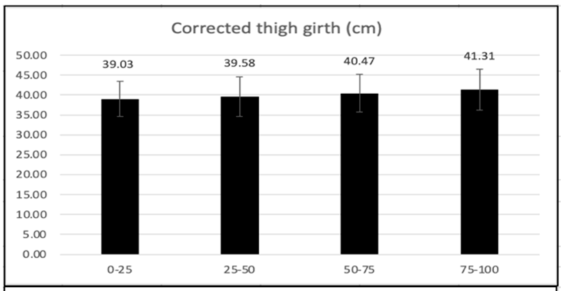

0-25 vs 25-50: Mean Diff: -0.555; p=1.000; 95% CI: -1.93; 0.82
0-25 vs 50-75: Mean Diff: -1.439; p=0.062; 95% CI: -2.92; 0.04
0-25 vs 75-100: Mean Diff: -2.281; p<0.001; 95% CI: -3.64; -0.92
25-50 vs 50-75: Mean Diff: -0.884; p=0.510; 95% CI: -2.24; 0.47
25-50 vs 75-100: Mean Diff: -1.726; p=0.001; 95% CI: -2.95; -0.50
50-75 vs 75-100: Mean Diff: -0.843; p=0.573; 95% CI: -2.18; 0.49

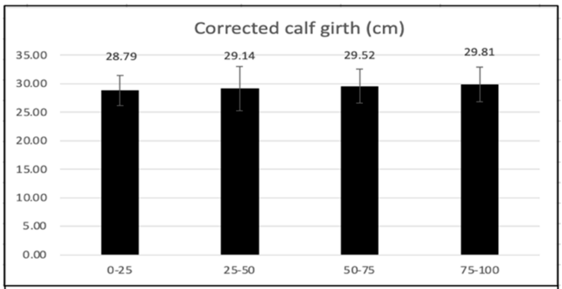

0-25 vs 25-50: Mean Diff: -0.354; p=1.000; 95% CI: -1.27; 0.56
0-25 vs 50-75: Mean Diff: -0.733; p=0.291; 95% CI: -1.72; 0.25
0-25 vs 75-100: Mean Diff: -1.024; p=0.017; 95% CI: -1.93; -0.12
25-50 vs 50-75: Mean Diff: -0.379; p=1.000; 95% CI: -1.28; 0.52
25-50 vs 75-100: Mean Diff: -0.670; p=0.175; 95% CI: -1.48; 0.14
50-75 vs 75-100: Mean Diff: -0.291; p=1.000; 95% CI: -1.18; 0.59

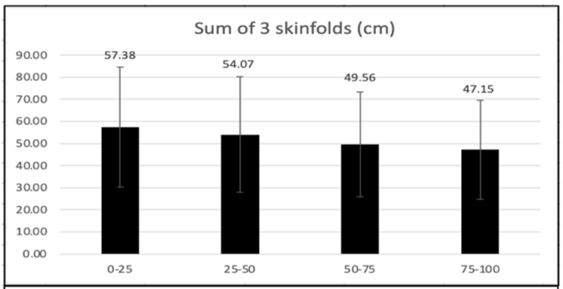

0-25 vs 25-50: Mean Diff: 3.317; p=1.000; 95% CI: -3.72; 10.35
0-25 vs 50-75: Mean Diff: 7.822; p=0.038; 95% CI: 0.27; 15.37
0-25 vs 75-100: Mean Diff: 10.234; p=0.001; 95% CI: 3.30; 17.17
25-50 vs 50-75: Mean Diff: 4.505; p=0.512; 95% CI: -2.41; 11.42
25-50 vs 75-100: Mean Diff: 6.917; p=0.021; 95% CI: 0.68; 13.16
50-75 vs 75-100: Mean Diff: 2.412; p=1.000; 95% CI: -4.40; 9.23

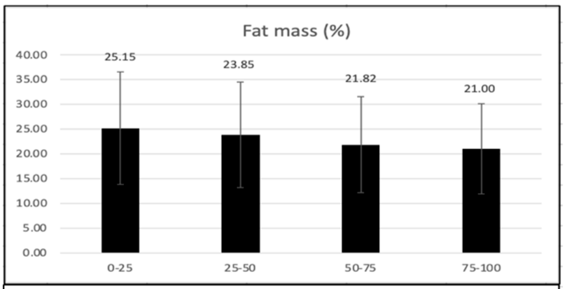

0-25 vs 25-50: Mean Diff: 1.303; p=1.000; 95% CI: -1.58; 4.19
0-25 vs 50-75: Mean Diff: 3.334; p=0.027; 95% CI: 0.24; 6.43
0-25 vs 75-100: Mean Diff: 4.157; p=0.001; 95% CI: 1.32; 7.00
25-50 vs 50-75: Mean Diff: 2.030; p=0.350; 95% CI: -0.80; 4.86
25-50 vs 75-100: Mean Diff: 2.854; p=0.019; 95% CI: 0.30; 5.41
50-75 vs 75-100: Mean Diff: 0.824; p=1.000; 95% CI: -1.97; 3.61

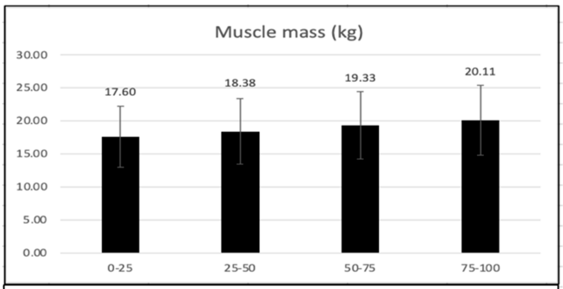

0-25 vs 25-50: Mean Diff: -0.785; p=0.874; 95% CI: -2.21; 0.64
0-25 vs 50-75: Mean Diff: -1.735; p=0.017; 95% CI: -3.27; -0.20
0-25 vs 75-100: Mean Diff: -2.507; p<0.001; 95% CI: -3.91; -1.10
25-50 vs 50-75: Mean Diff: -0.949; p=0.442; 95% CI: -2.35; 0.45
25-50 vs 75-100: Mean Diff: -1.721; p=0.002; 95% CI: -2.99; -0.46
50-75 vs 75-100: Mean Diff: -0.772; p=0.838; 95% CI: -2.15; 0.61

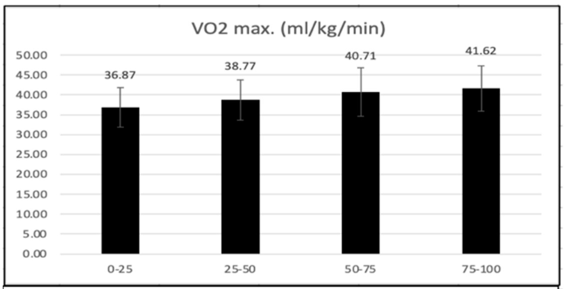

0-25 vs 25-50: Mean Diff: -1.906; p=0.007; 95% CI: -3.45; -0.36
0-25 vs 50-75: Mean Diff: -3.837; p<0.001; 95% CI: -5.49; -2.18
0-25 vs 75-100: Mean Diff: -4.753; p<0.001; 95% CI: -6.27; -3.23
25-50 vs 50-75: Mean Diff: -1.932; p=0.005; 95% CI: -3.45; -0.41
25-50 vs 75-100: Mean Diff: -2.847; p<0.001; 95% CI: -4.22; -1.48
50-75 vs 75-100: Mean Diff: -0.916; p=0.633; 95% CI: -2.41; 0.58

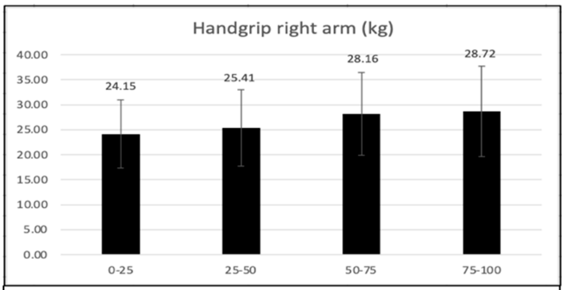

0-25 vs 25-50: Mean Diff: -1.262; p=0.878; 95% CI: -3.56; 1.03
0-25 vs 50-75: Mean Diff: -4.014; p<0.001; 95% CI: -6.48; -1.55
0-25 vs 75-100: Mean Diff: -4.575; p<0.001; 95% CI: -6.84; -2.31
25-50 vs 50-75: Mean Diff: -2.752; p=0.008; 95% CI: -5.01; -0.50
25-50 vs 75-100: Mean Diff: -3.312; p<0.001; 95% CI: -5.35; -1.28
50-75 vs 75-100: Mean Diff: -0.560; p=1.000; 95% CI: -2.78; 1.66

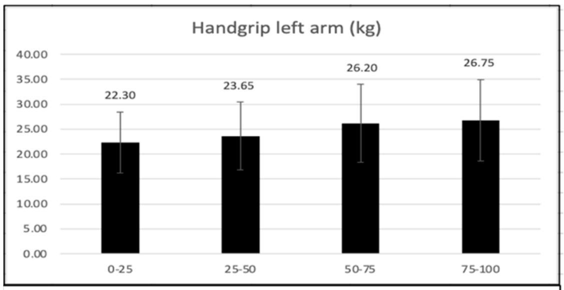

0-25 vs 25-50: Mean Diff: -1.352; p=0.521; 95% CI: -3.44; 0.73
0-25 vs 50-75: Mean Diff: -3.902; p<0.001; 95% CI: -6.14; -1.66
0-25 vs 75-100: Mean Diff: -4.448; p<0.001; 95% CI: -6.50; -2.39
25-50 vs 50-75: Mean Diff: -2.550; p=0.006; 95% CI: -4.60; -0.50
25-50 vs 75-100: Mean Diff: -3.096; p<0.001; 95% CI: -4.95; -1.25
50-75 vs 75-100: Mean Diff: -0.546; p=1.000; 95% CI: -2.57; 1.47

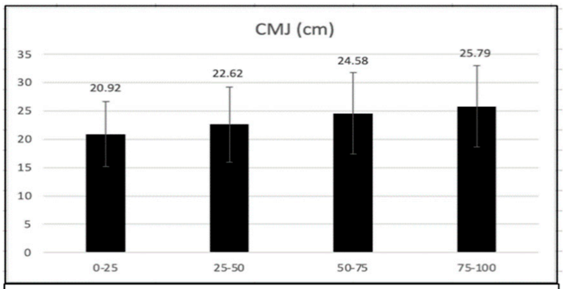

0-25 vs 25-50: Mean Diff: -1.698; p=0.115; 95% CI: -3.61; 0.22
0-25 vs 50-75: Mean Diff: -3.662; p<0.001; 95% CI: -5.72; -1.61
0-25 vs 75-100: Mean Diff: -4.875; p<0.001; 95% CI: -6.76; -2.99
25-50 vs 50-75: Mean Diff: -1.964; p=0.035; 95% CI: -3.84; -0.08
25-50 vs 75-100: Mean Diff: -3.177; p<0.001; 95% CI: -4.87; -1.48
50-75 vs 75-100: Mean Diff: -1.214; p=0.502; 95% CI: -3.07; 0.64

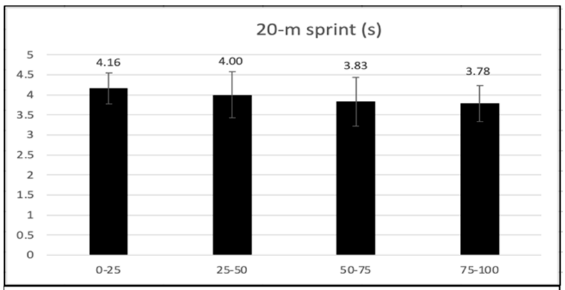

0-25 vs 25-50: Mean Diff: 0.158; p=0.026; 95% CI: 0.01; 0.30
0-25 vs 50-75: Mean Diff: 0.331; p<0.001; 95% CI: 0.17; 0.49
0-25 vs 75-100: Mean Diff: 0.378; p<0.001; 95% CI: 0.23; 0.52
25-50 vs 50-75: Mean Diff: 0.173; p=0.009; 95% CI: 0.03; 0.32
25-50 vs 75-100: Mean Diff: 0.220; p<0.001; 95% CI: 0.09; 0.35
50-75 vs 75-100: Mean Diff: 0.047; p=1.000; 95% CI: -0.09; 0.19

Figure S1. Bonferroni post-hoc analysis of the variables that showed significant differences in competence.
