# Supplementary material for: The Role of Basic Psychological Needs in the Adoption of Healthy Habits by Adolescents
Source: Behav Sci (Basel). 2023 Jul 14;13(7):592. doi: 10.3390/bs13070592 (PMC10376858; doi:10.3390/bs13070592)
Supplement: Supplementary file 1 [file behavsci-13-00592-s001.zip › Supplementary Figure S2.pdf]

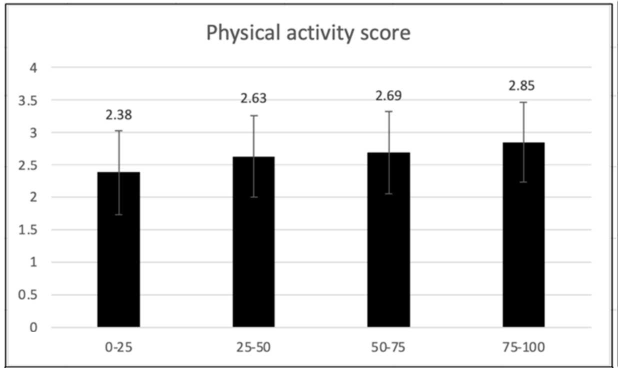

0-25 vs 25-50: Mean Diff: -0.251; p=0.002; 95% CI: -0.43; -0.07
0-25 vs 50-75: Mean Diff: -0.308; p<0.001; 95% CI: -0.48; -0.13
0-25 vs 75-100: Mean Diff: -0.469; p<0.001; 95% CI: -0.64; -0.30
25-50 vs 50-75: Mean Diff: -0.057; p=1.000; 95% CI: -0.24; 0.12
25-50 vs 75-100: Mean Diff: -0.218; p=0.006; 95% CI: -0.39; -0.04
50-75 vs 75-100: Mean Diff: -0.161; p=0.060; 95% CI: -0.33; 0.01

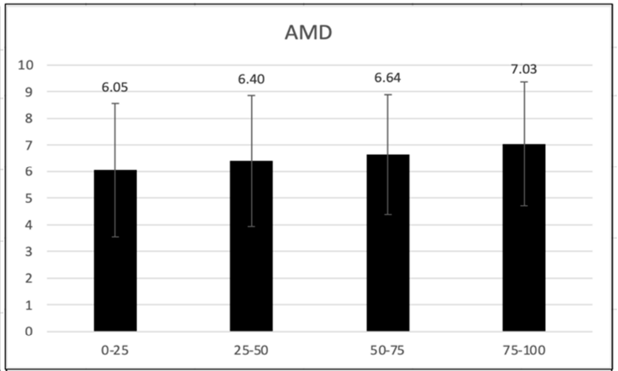

0-25 vs 25-50: Mean Diff: -0.35; p=1.000; 95% CI: -1.04; 0.34
0-25 vs 50-75: Mean Diff: -0.59; p=0.102; 95% CI: -1.24; 0.06
0-25 vs 75-100: Mean Diff: -0.98; p<0.001; 95% CI: -1.62; -0.34
25-50 vs 50-75: Mean Diff: -0.24; p=1.000; 95% CI: -0.92; 0.43
25-50 vs 75-100: Mean Diff: -0.63; p=0.068; 95% CI: -1.30; 0.03
50-75 vs 75-100: Mean Diff: -0.39; p=0.567; 95% CI: -1.01; 0.23

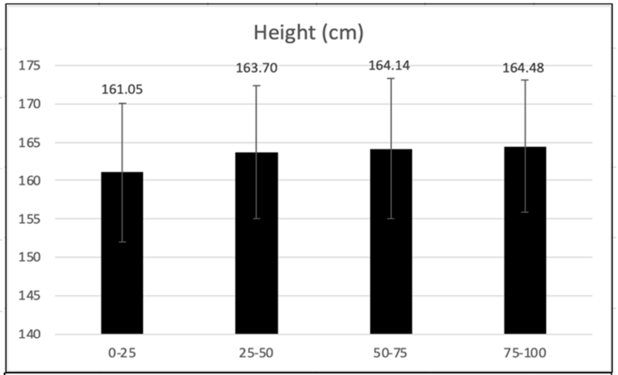

0-25 vs 25-50: Mean Diff: -2.654; p=0.040; 95% CI: -5.23; -0.08
0-25 vs 50-75: Mean Diff: -3.095; p=0.005; 95% CI: -5.53; -0.66
0-25 vs 75-100: Mean Diff: -3.435; p=0.001; 95% CI: -5.81; -1.06
25-50 vs 50-75: Mean Diff: -0.441; p=1.000; 95% CI: -2.96; 2.08
25-50 vs 75-100: Mean Diff: -0.782; p=1.000; 95% CI: -3.25; 1.69
50-75 vs 75-100: Mean Diff: -0.341; p=1.000; 95% CI: -2.66; 1.98

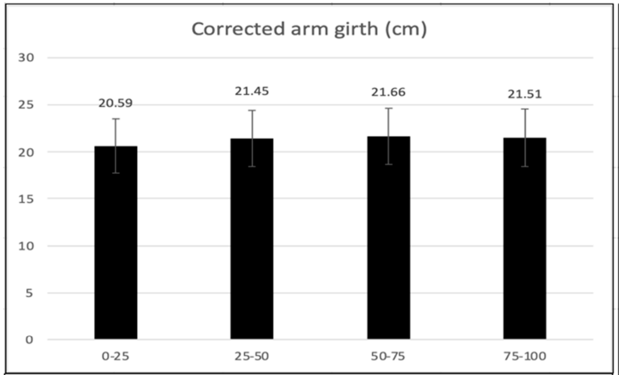

0-25 vs 25-50: Mean Diff: -0.858; p=0.053; 95% CI: -1.72; 0.01
0-25 vs 50-75: Mean Diff: -1.064; p=0.004; 95% CI: -1.88; -0.25
0-25 vs 75-100: Mean Diff: -0.919; p=0.015; 95% CI: -1.72; -0.12
25-50 vs 50-75: Mean Diff: -0.206; p=1.000; 95% CI: -1.05; 0.64
25-50 vs 75-100: Mean Diff: -0.061; p=1.000; 95% CI: -0.89; 0.77
50-75 vs 75-100: Mean Diff: 0.145; p=1.000; 95% CI: -0.63; 0.92

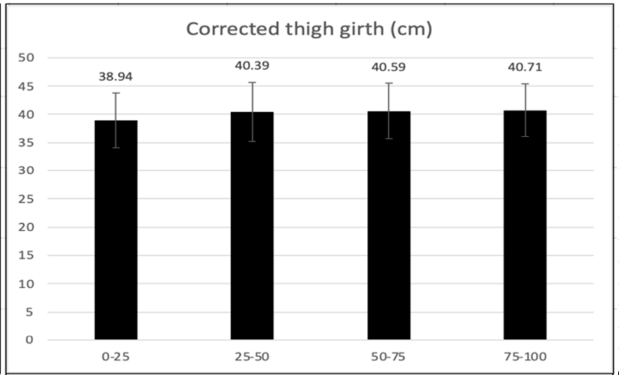

0-25 vs 25-50: Mean Diff: -1.450; p=0.042; 95% CI: -2.87; -0.03
0-25 vs 50-75: Mean Diff: -1.650; p=0.007; 95% CI: -2.99; -0.31
0-25 vs 75-100: Mean Diff: -1.762; p=0.002; 95% CI: -3.07; -0.45
25-50 vs 50-75: Mean Diff: -0.200; p=1.000; 95% CI: -1.59; 1.19
25-50 vs 75-100: Mean Diff: -0.312; p=1.000; 95% CI: -1.67; 1.05
50-75 vs 75-100: Mean Diff: -0.112; p=1.000; 95% CI: -1.39; 1.16

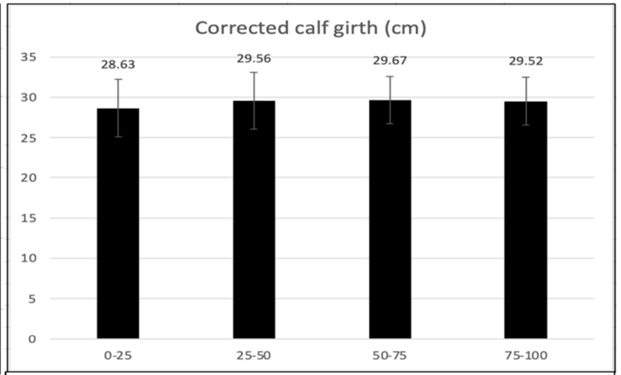

0-25 vs 25-50: Mean Diff: -0.936; p=0.050; 95% CI: -1.87; 0.01
0-25 vs 50-75: Mean Diff: -1.050; p=0.010; 95% CI: -1.93; -0.17
0-25 vs 75-100: Mean Diff: -0.894; p=0.038; 95% CI: -1.76; -0.03
25-50 vs 50-75: Mean Diff: -0.114; p=1.000; 95% CI: -1.03; 0.80
25-50 vs 75-100: Mean Diff: 0.041; p=1.000; 95% CI: -0.86; 0.94
50-75 vs 75-100: Mean Diff: 0.156; p=1.000; 95% CI: -0.68; 1.00

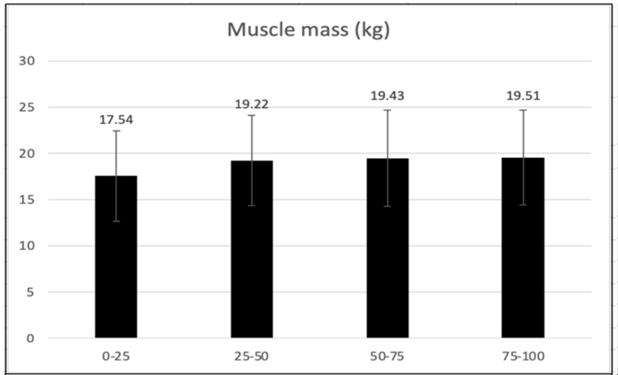

0-25 vs 25-50: Mean Diff: -1.682; p=0.015; 95% CI: -3.15; -0.21
0-25 vs 50-75: Mean Diff: -1.884; p=0.002; 95% CI: -3.27; -0.50
0-25 vs 75-100: Mean Diff: -1.967; p=0.001; 95% CI: -3.32; -0.61
25-50 vs 50-75: Mean Diff: -0.202; p=1.000; 95% CI: -1.64; 1.23
25-50 vs 75-100: Mean Diff: -0.285; p=1.000; 95% CI: -1.69; 1.12
50-75 vs 75-100: Mean Diff: -0.082; p=1.000; 95% CI: -1.40; 1.24

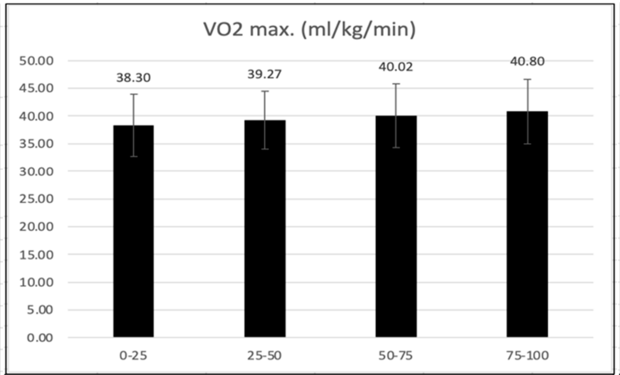

0-25 vs 25-50: Mean Diff: -0.970; p=0.711; 95% CI: -2.61; 0.67
0-25 vs 50-75: Mean Diff: -1.713; p=0.021; 95% CI: -3.26; -0.17
0-25 vs 75-100: Mean Diff: -2.492; p<0.001; 95% CI: -4.01; -0.98
25-50 vs 50-75: Mean Diff: -0.743; p=1.000; 95% CI: -2.35; 0.86
25-50 vs 75-100: Mean Diff: -1.522; p=0.064; 95% CI: -3.09; 0.05
50-75 vs 75-100: Mean Diff: -0.779; p=0.975; 95% CI: -2.25; 0.70

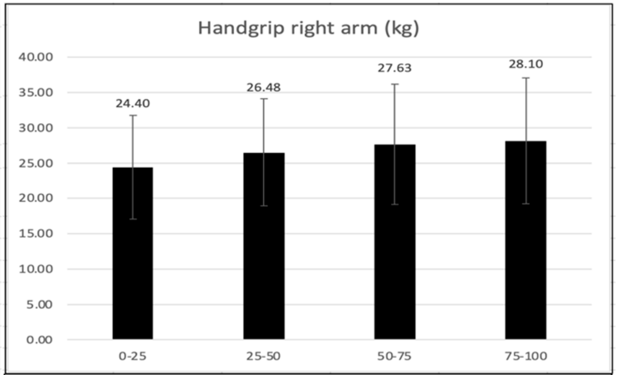

0-25 vs 25-50: Mean Diff: -2.076; p=0.127; 95% CI: -4.45; 0.30
0-25 vs 50-75: Mean Diff: -3.228; p=0.001; 95% CI: -5.47; -0.99
0-25 vs 75-100: Mean Diff: -3.693; p<0.001; 95% CI: -5.89; -1.50
25-50 vs 50-75: Mean Diff: -1.152; p=1.000; 95% CI: -3.47; 1.17
25-50 vs 75-100: Mean Diff: -1.617; p=0.364; 95% CI: -3.89; 0.66
50-75 vs 75-100: Mean Diff: -0.465; p=1.000; 95% CI: -2.60; 1.67

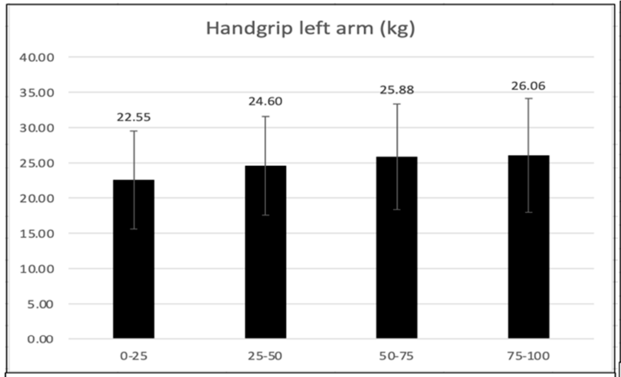

0-25 vs 25-50: Mean Diff: -2.052; p=0.073; 95% CI: -4.21; 0.11
0-25 vs 50-75: Mean Diff: -3.327; p<0.001; 95% CI: -5.36; -1.29
0-25 vs 75-100: Mean Diff: -3.515; p<0.001; 95% CI: -5.51; -1.52
25-50 vs 50-75: Mean Diff: -1.274; p=0.664; 95% CI: -3.38; 0.84
25-50 vs 75-100: Mean Diff: -1.462; p=0.372; 95% CI: -3.53; 0.61
50-75 vs 75-100: Mean Diff: -0.188; p=1.000; 95% CI: -2.13; 1.75

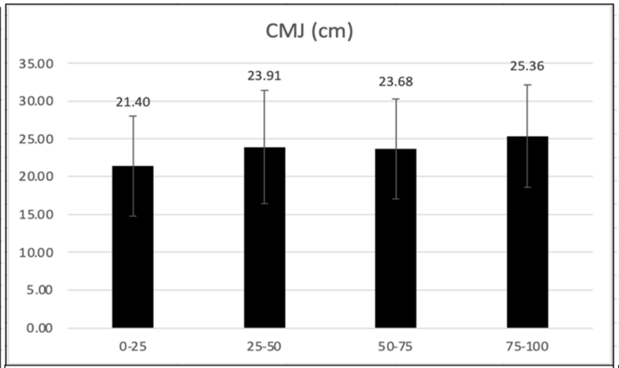

0-25 vs 25-50: Mean Diff: -2.513; p=0.005; 95% CI: -4.50; -0.53
0-25 vs 50-75: Mean Diff: -2.288; p=0.008; 95% CI: -4.16; -0.41
0-25 vs 75-100: Mean Diff: -3.961; p<0.001; 95% CI: -5.79; -2.13
25-50 vs 50-75: Mean Diff: 0.225; p=1.000; 95% CI: -1.72; 2.17
25-50 vs 75-100: Mean Diff: -1.448; p=0.268; 95% CI: -3.35; 0.46
50-75 vs 75-100: Mean Diff: -1.673; p=0.080; 95% CI: -3.46; 0.11

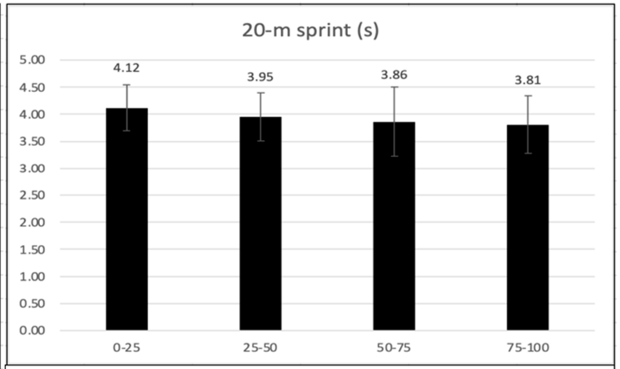

0-25 vs 25-50: Mean Diff: 0.175; p=0.014; 95% CI: 0.02; 0.33
0-25 vs 50-75: Mean Diff: 0.266; p<0.001; 95% CI: 0.12; 0.41
0-25 vs 75-100: Mean Diff: 0.309; p<0.001; 95% CI: 0.17; 0.45
25-50 vs 50-75: Mean Diff: 0.091; p=0.617; 95% CI: -0.06; 0.24
25-50 vs 75-100: Mean Diff: 0.134; p=0.089; 95% CI: -0.01; 0.28
50-75 vs 75-100: Mean Diff: 0.042; p=1.000; 95% CI: -0.09; 0.18

Figure S2. Bonferroni post-hoc analysis of the variables that showed significant differences in autonomy.
