# Supplementary material for: The Role of Basic Psychological Needs in the Adoption of Healthy Habits by Adolescents
Source: Behav Sci (Basel). 2023 Jul 14;13(7):592. doi: 10.3390/bs13070592 (PMC10376858; doi:10.3390/bs13070592)
Supplement: Supplementary file 1 [file behavsci-13-00592-s001.zip › Supplementary Figure S3.pdf]

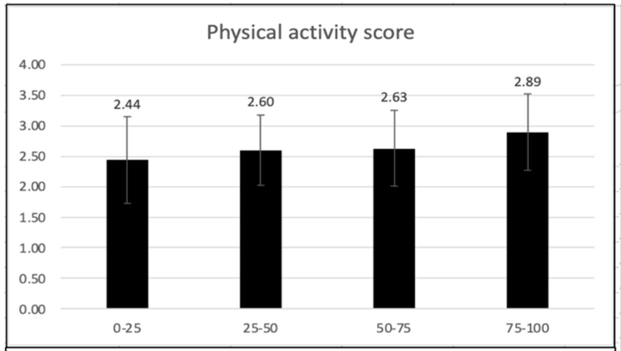

0-25 vs 25-50: Mean Diff: -0.155; p=0.154; 95% CI: -0.34; 0.03
0-25 vs 50-75: Mean Diff: -0.189; p=0.026; 95% CI: -0.36; -0.01
0-25 vs 75-100: Mean Diff: -0.445; p<0.001; 95% CI: -0.62; -0.27
25-50 vs 50-75: Mean Diff: -0.034; p=1.000; 95% CI: -0.21; 0.14
25-50 vs 75-100: Mean Diff: -0.289; p<0.001; 95% CI: -0.46; -0.11
50-75 vs 75-100: Mean Diff: -0.255; p<0.001; 95% CI: -0.42; -0.09

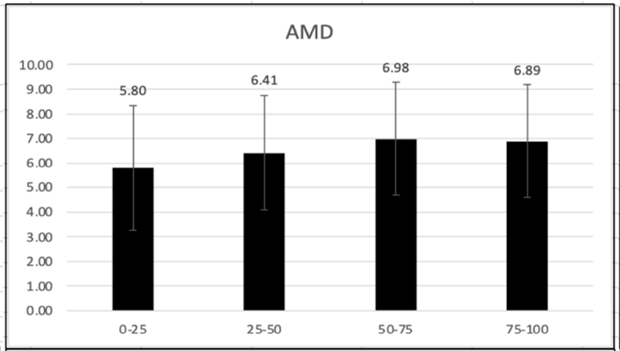

0-25 vs 25-50: Mean Diff: -0.62; p=0.104; 95% CI: -1.30; 0.07
0-25 vs 50-75: Mean Diff: -1.18; p<0.001; 95% CI: -1.83; -0.53
0-25 vs 75-100: Mean Diff: -1.09; p<0.001; 95% CI: -1.74; -0.44
25-50 vs 50-75: Mean Diff: -0.56; p=0.135; 95% CI: -1.21; 0.09
25-50 vs 75-100: Mean Diff: -0.48; p=0.321; 95% CI: -1.13; 0.17
50-75 vs 75-100: Mean Diff: 0.09; p=1.000; 95% CI: -0.53; 0.70

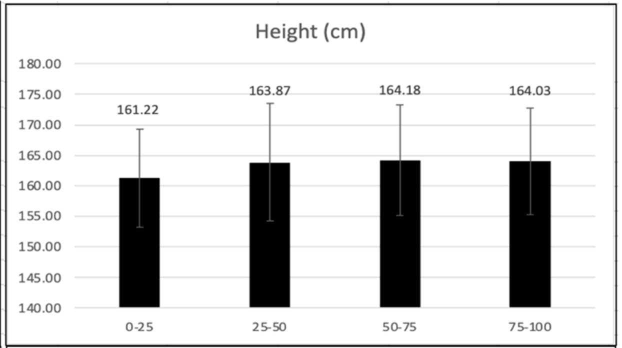

0-25 vs 25-50: Mean Diff: -2.651; p=0.040; 95% CI: -5.23; -0.07
0-25 vs 50-75: Mean Diff: -2.965; p=0.009; 95% CI: -5.42; -0.51
0-25 vs 75-100: Mean Diff: -2.816; p=0.015; 95% CI: -5.27; -0.36
25-50 vs 50-75: Mean Diff: -0.315; p=1.000; 95% CI: -2.77; 2.14
25-50 vs 75-100: Mean Diff: -0.166; p=1.000; 95% CI: -2.62; 2.29
50-75 vs 75-100: Mean Diff: 0.149; p=1.000; 95% CI: -2.17; 2.47

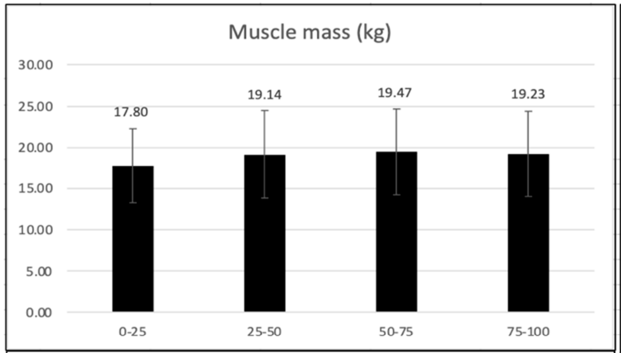

0-25 vs 25-50: Mean Diff: -1.345; p=0.095; 95% CI: -2.82; 0.13
0-25 vs 50-75: Mean Diff: -1.675; p=0.010; 95% CI: -3.08; -0.27
0-25 vs 75-100: Mean Diff: -1.434; p=0.041; 95% CI: -2.83; -0.04
25-50 vs 50-75: Mean Diff: -0.330; p=1.000; 95% CI: -1.73; 1.07
25-50 vs 75-100: Mean Diff: -0.088; p=1.000; 95% CI: -1.49; 1.31
50-75 vs 75-100: Mean Diff: 0.241; p=1.000; 95% CI: -1.08; 1.56

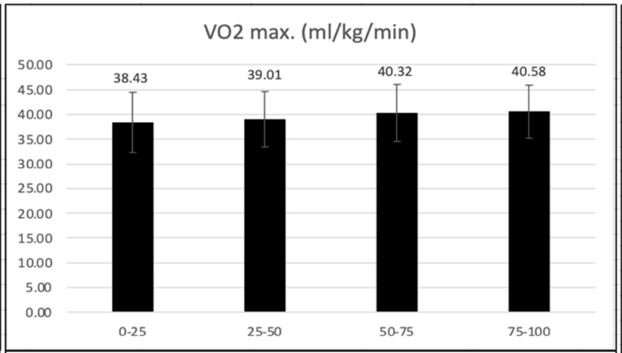

0-25 vs 25-50: Mean Diff: -0.578; p=1.000; 95% CI: -2.22; 1.06
0-25 vs 50-75: Mean Diff: -1.890; p=0.008; 95% CI: -3.45; -0.33
0-25 vs 75-100: Mean Diff: -2.148; p=0.002; 95% CI: -3.71; -0.59
25-50 vs 50-75: Mean Diff: -1.311; p=0.159; 95% CI: -2.87; 0.25
25-50 vs 75-100: Mean Diff: -1.569; p=0.047; 95% CI: -3.13; -0.01
50-75 vs 75-100: Mean Diff: -0.258; p=1.000; 95% CI: -1.73; 1.22

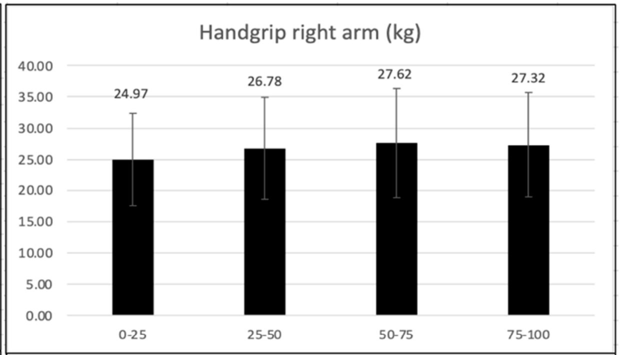

0-25 vs 25-50: Mean Diff: -1.803; p=0.278; 95% CI: -4.19; 0.59
0-25 vs 50-75: Mean Diff: -2.644; p=0.013; 95% CI: -4.92; -0.37
0-25 vs 75-100: Mean Diff: -2.342; p=0.039; 95% CI: -4.61; -0.07
25-50 vs 50-75: Mean Diff: -0.841; p=1.000; 95% CI: -3.11; 1.43
25-50 vs 75-100: Mean Diff: -0.538; p=1.000; 95% CI: -2.81; 1.73
50-75 vs 75-100: Mean Diff: 0.303; p=1.000; 95% CI: -1.85; 2.45

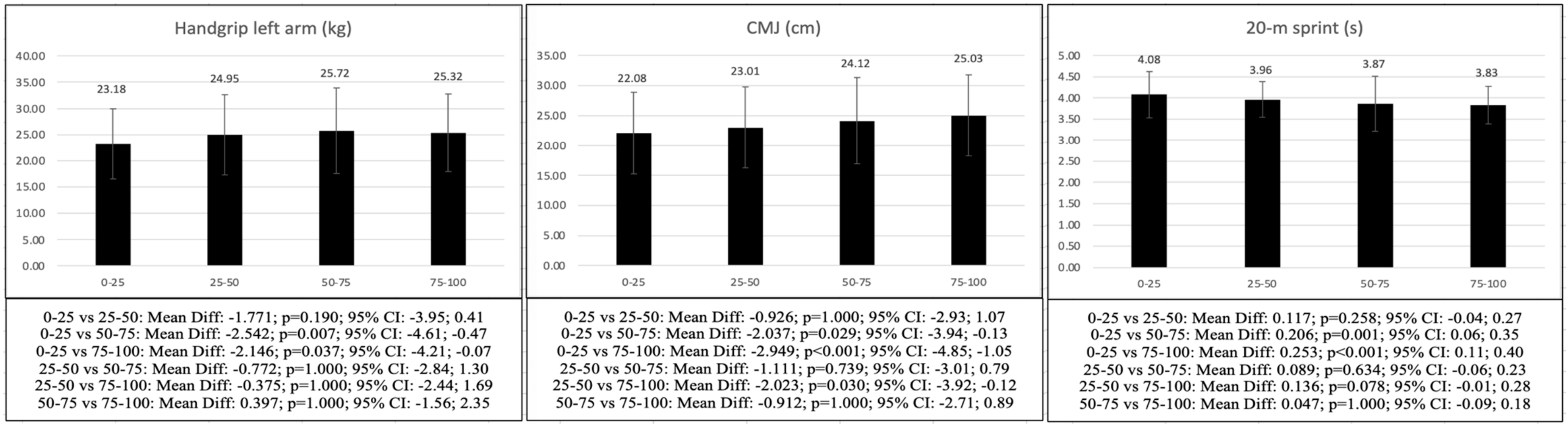

Handgrip left arm (kg)

0-25 vs 25-50: Mean Diff: -1.771; p=0.190; 95% CI: -3.95; 0.41
0-25 vs 50-75: Mean Diff: -2.542; p=0.007; 95% CI: -4.61; -0.47
0-25 vs 75-100: Mean Diff: -2.146; p=0.037; 95% CI: -4.21; -0.07
25-50 vs 50-75: Mean Diff: -0.772; p=1.000; 95% CI: -2.84; 1.30
25-50 vs 75-100: Mean Diff: -0.375; p=1.000; 95% CI: -2.44; 1.69
50-75 vs 75-100: Mean Diff: 0.397; p=1.000; 95% CI: -1.56; 2.35

CMJ (cm)

0-25 vs 25-50: Mean Diff: -0.926; p=1.000; 95% CI: -2.93; 1.07
0-25 vs 50-75: Mean Diff: -2.037; p=0.029; 95% CI: -3.94; -0.13
0-25 vs 75-100: Mean Diff: -2.949; p<0.001; 95% CI: -4.85; -1.05
25-50 vs 50-75: Mean Diff: -1.111; p=0.739; 95% CI: -3.01; 0.79
25-50 vs 75-100: Mean Diff: -2.023; p=0.030; 95% CI: -3.92; -0.12
50-75 vs 75-100: Mean Diff: -0.912; p=1.000; 95% CI: -2.71; 0.89

20-m sprint (s)

0-25 vs 25-50: Mean Diff: 0.117; p=0.258; 95% CI: -0.04; 0.27
0-25 vs 50-75: Mean Diff: 0.206; p=0.001; 95% CI: 0.06; 0.35
0-25 vs 75-100: Mean Diff: 0.253; p<0.001; 95% CI: 0.11; 0.40
25-50 vs 50-75: Mean Diff: 0.089; p=0.634; 95% CI: -0.06; 0.23
25-50 vs 75-100: Mean Diff: 0.136; p=0.078; 95% CI: -0.01; 0.28
50-75 vs 75-100: Mean Diff: 0.047; p=1.000; 95% CI: -0.09; 0.18

Figure S3. Bonferroni post-hoc analysis of the variables that showed significant differences in relatedness.
