# Supplementary material for: The Role of Basic Psychological Needs in the Adoption of Healthy Habits by Adolescents
Source: Behav Sci (Basel). 2023 Jul 14;13(7):592. doi: 10.3390/bs13070592 (PMC10376858; doi:10.3390/bs13070592)
Supplement: Supplementary file 1 [file behavsci-13-00592-s001.zip › Supplementary Figure S4.pdf]

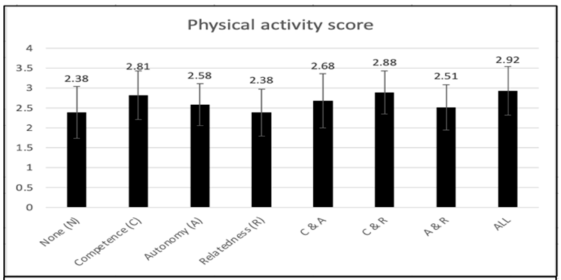


N vs C: Mean Diff: -0.433; p=0.005; 95% CI: -0.79; -0.07
N vs C & A: Mean Diff: -0.306; p=0.010; 95% CI: -0.57; -0.04
N vs C & R: Mean Diff: -0.505; p<0.001; 95% CI: -0.81; -0.20
N vs ALL: Mean Diff: -0.542; p<0.001; 95% CI: -0.74; -0.35
C vs R: Mean Diff: 0.427; p=0.023; 95% CI: 0.03; 0.83
C & R vs A & R: Mean Diff: 0.375; p=0.039; 95% CI: 0.01; 0.74
ALL vs A: Mean Diff: 0.342; p=0.009; 95% CI: 0.05; 0.64
ALL vs R: Mean Diff: 0.536; p<0.001; 95% CI: 0.28; 0.79
ALL vs A & R: Mean Diff: 0.413; p<0.001; 95% CI: 0.13; 0.69

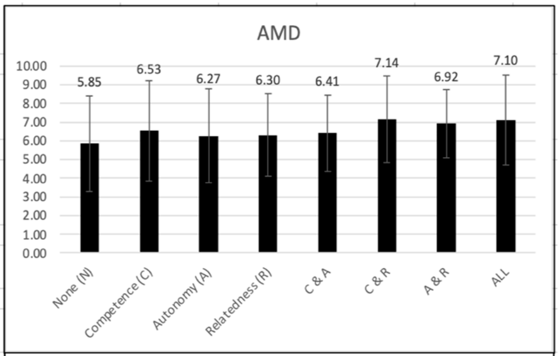


N vs C & R: Mean Diff: -1.29; p=0.017; 95% CI: -2.47; -0.11
N vs ALL: Mean Diff: -1.25; p<0.001; 95% CI: -1.99; -0.51

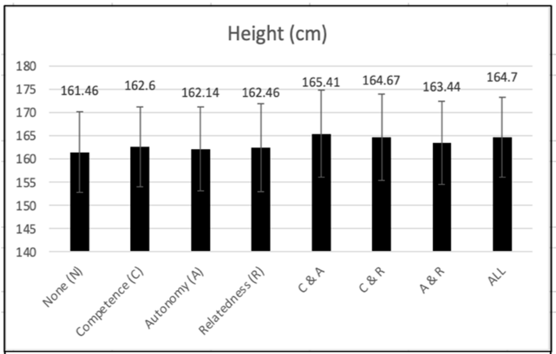


N vs C & A: Mean Diff: -3.954; p=0.041; 95% CI: -7.84; -0.07
N vs ALL: Mean Diff: -3.239; p=0.009; 95% CI: -6.04; -0.44

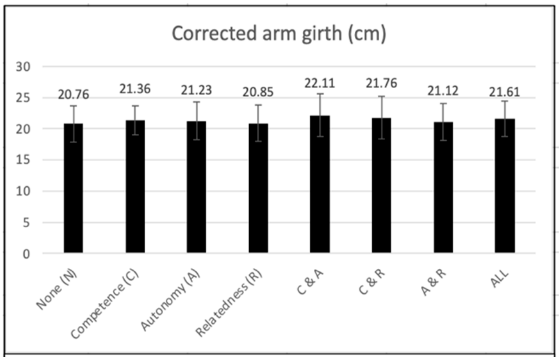


N vs C & A: Mean Diff: -1.350; p=0.034; 95% CI: -2.65; -0.05

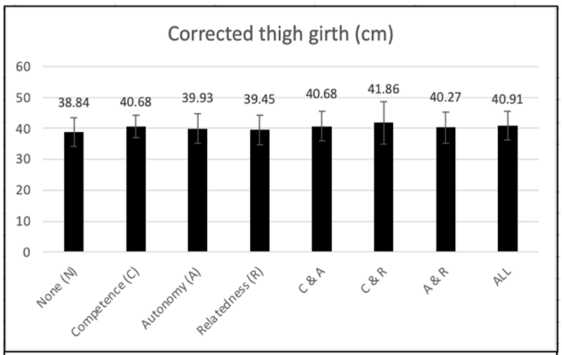


N vs C & R: Mean Diff: -3.02; p=0.003; 95% CI: -5.45; -0.59
N vs ALL: Mean Diff: -2.070; p=0.001; 95% CI: -3.60; -0.54

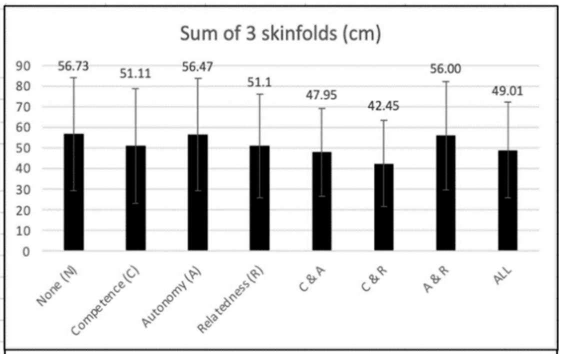


N vs C & R: Mean Diff: 14.284; p=0.009; 95% CI: 1.89; 26.67

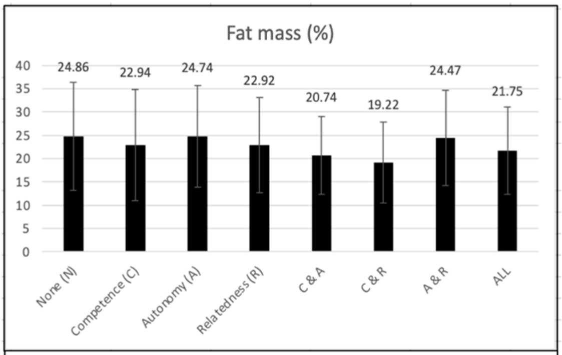


N vs C & R: Mean Diff: 5.646; p=0.014; 95% CI: 0.57; 10.72

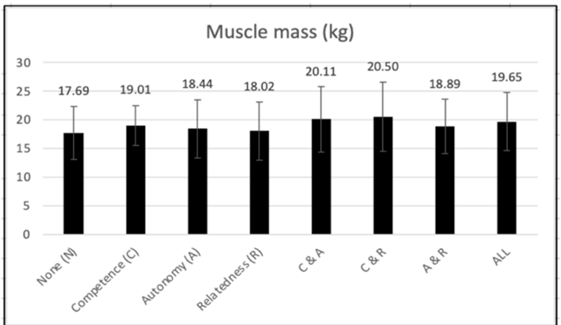


N vs C & A: Mean Diff: -2.418; p=0.017; 95% CI: -4.62; -0.21
N vs C & R: Mean Diff: -2.813; p=0.014; 95% CI: -5.33; -0.30
N vs ALL: Mean Diff: -1.960; p=0.003; 95% CI: -3.55; -0.37

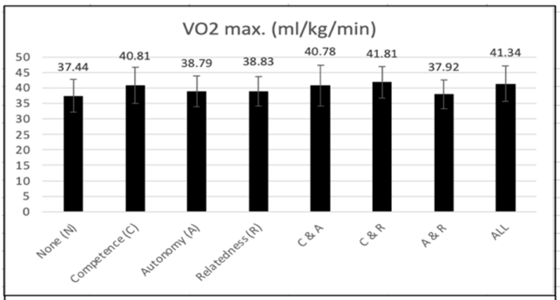


N vs C: Mean Diff: -3.372; p=0.031; 95% CI: -6.60; -0.15
N vs C & A: Mean Diff: -3.345; p<0.001; 95% CI: -5.74; -0.95
N vs C & R: Mean Diff: -4.371; p<0.001; 95% CI: -7.11; -1.63
N vs ALL: Mean Diff: -3.904; p<0.001; 95% CI: -5.63; -2.17
C & R vs A & R: Mean Diff: 3.890; p=0.006; 95% CI: 0.61; 7.17
A & R vs ALL: Mean Diff: -3.422; p=0.001; 95% CI: -5.92; -0.92

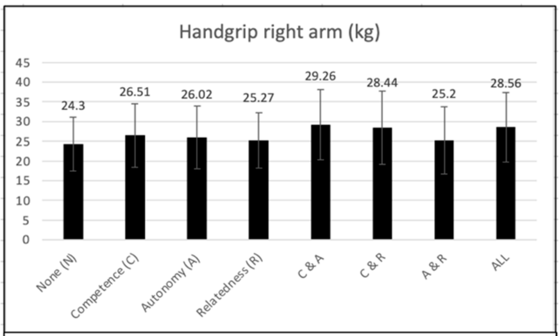


N vs C & A: Mean Diff: -4.956; p<0.001; 95% CI: -8.50; -1.41
N vs C & R: Mean Diff: -4.143; p=0.039; 95% CI: -8.19; -0.10
N vs ALL: Mean Diff: -4.259; p<0.001; 95% CI: -6.81; -1.70

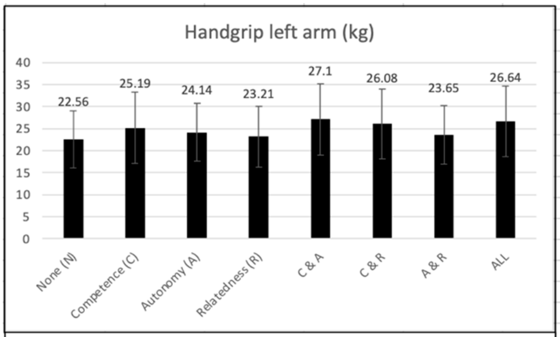


N vs C & A: Mean Diff: -4.543; p<0.001; 95% CI: -7.77; -1.32
N vs ALL: Mean Diff: -4.085; p<0.001; 95% CI: -6.41; -1.76

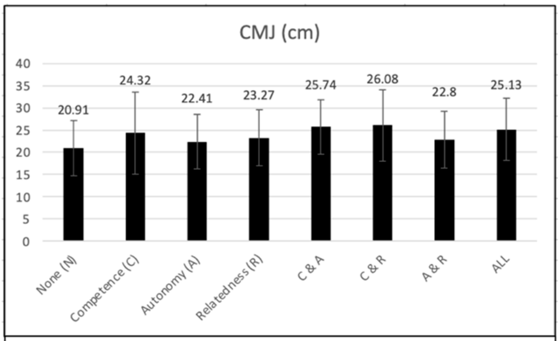


N vs C & A: Mean Diff: -4.832; p<0.001; 95% CI: -7.79; -1.88
N vs C & R: Mean Diff: -5.167; p<0.001; 95% CI: -8.54; -1.79
N vs ALL: Mean Diff: -4.223; p<0.001; 95% CI: -6.35; -2.09

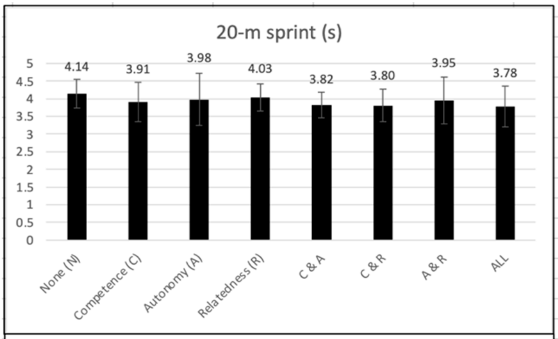


N vs C & A: Mean Diff: 0.326; p<0.001; 95% CI: 0.10; 0.55
N vs C & R: Mean Diff: 0.348; p=0.001; 95% CI: 0.09; 0.61
N vs ALL: Mean Diff: 0.365; p<0.001; 95% CI: 0.20; 0.53

Figure S4. Bonferroni post hoc of the variables that showed differences according to the psychological needs satisfied by the adolescents.
